# Value of Ki-67 expression in triple-negative breast cancer before and after neoadjuvant chemotherapy with weekly paclitaxel plus carboplatin

**DOI:** 10.1038/srep30091

**Published:** 2016-07-18

**Authors:** Ruo-Xi Wang, Sheng Chen, Xi Jin, Zhi-Ming Shao

**Affiliations:** 1 Department of Breast Surgery, Fudan University Shanghai Cancer Center/Cancer Institute, Shanghai, P.R. China; 2 Department of Oncology, Shanghai Medical College, Fudan University, Shanghai, P.R. China; 3 Institutes of Biomedical Science, Fudan University, Shanghai, P.R. China

## Abstract

Neoadjuvant chemotherapy (NCT) is one of the main treatment strategies for patients with locally advanced breast cancer. In this study, we focused on the predictive and prognostic value of Ki-67 in triple-negative breast cancer (TNBC) patients who received NCT. Data from 280 patients with stage II–III TNBC were collected. All patients were treated according to the same protocol with weekly paclitaxel and carboplatin. The overall pCR rate was 33.9%. Both the categorical and linear Ki-67 were independently correlated with pCR (P < 0.001). There were also statistically significant differences among Ki-67 categories with respect to clinical response (P < 0.001), Miller-Payne (MP) grades (P < 0.001), and node status (P < 0.001). A significant reduction of Ki-67 after NCT was most likely observed in patients with a relatively better response. In the multivariate model for non-pCR patients, Ki-67 reduction presented an independent prognostic value for relapse of disease (HR = 0.986, 95% CI: 0.978–0.994; P = 0.001). This study had indicated that the primary Ki-67 might help in further classifying TNBCs into subtypes with different responses to chemotherapy and a significant reduction of Ki-67 after treatment could indicate a favorable prognosis in non-pCR patients.

Breast cancers have been classified into four subtypes (luminal A, luminal B, HER-2 positive, and triple-negative) according to the status of hormonal receptors, Ki-67 and human epidermal growth factor receptor-2 (HER-2)expression^1^. The characteristic feature of triple-negative breast cancer (TNBC) is that it lacks expression of estrogen, progesterone and human epidermal growth factor receptor-2/neu receptors. They comprise 15–20% of all breast cancers and have an aggressive tumor biology^2^.

Neoadjuvant chemotherapy (NCT) for TNBC has recently generated a growing interest because of the more aggressive biologic characteristics of this subtype and the lack of approved targeted therapies^3^. Systemic chemotherapy represents the mainstay of treatment for TNBC. Although TNBCs have relatively higher response rates compared with non-TNBCs^4^ and the pathological complete response predicts long-term outcome, most patients have residual disease with a higher risk of relapse^5^,^6^. This paradox suggests that TNBC is a heterogeneous group comprising subtypes with different clinical outcomes. With the exception of gene phenotype, several pathological biomarkers are used to identify subgroups of TNBC, including P53, cytokeratin (CK) 5/6, CK14, epidermal growth factor receptor (EGFR), and Ki-67^7^,^8^.

The proliferation marker Ki-67 has repeatedly been confirmed as an independent predictive and prognostic factor in early breast cancer^9^ and in the neoadjuvant setting^10^,^11^,^12^. Breast cancer with high Ki-67 expression responds better to chemotherapy but is associated with poor prognosis^10^,^11^,^12^, which is a similar situation to the TNBC paradox. Because the Ki-67 index is relatively higher in TNBC than in non-TNBC^13^, it might play an important role in clinical practice in classifying TNBCs with different biological behaviors.

Platinum agents, such as cisplatin and carboplatin, are DNA-damaging agents that have shown activity in breast cancer^14^. The use of platinum agents, in addition to standard NCT, has potential advantages particularly in the TNBC subgroup of breast cancer. This study was designed to demonstrate the utility of a platinum-containing treatment regimen in the neoadjuvant setting and to identify the predictive or prognostic value of Ki-67 among patients with TNBC.

## Results

### Characteristics and phredictors of pCR

A total of 280 patients with TNBC were enrolled in this study. The characteristics of the patients are shown in Table 1. Among 280 patients, the median Ki-67 value was 30%. Patients were classified into low-level (<20%), median-level (20%–50%), or high-level (>50%) Ki-67 groups according to the tertile cutoff points. Positive CK5/6 expression was found in 57.1% of tumors, and positive EGFR expression was found in 53.5% of tumors. Of 280 patients, 95 patients (33.9%) experienced pCR after completion of NCT, and a relatively higher proportion of pCR was observed in the high-level Ki-67 group. The primary tumor size and cancer stage were also correlated with the pCR. However, the basal-phenotype markers CK5/6 and EGFR failed to present predictive values. Table 1 shows the results of a Chi-squared test and multivariate logistic regression analysis for pCR predictors. The Ki-67 tertile and the Ki-67 linear value were independently correlated with the pCR (P < 0.001 and P < 0.001). The hazard ratios (HRs) of the Ki-67 tertiles were 2.490 for the median-level Ki-67 tertile (95% CI: 1.166–5.319) and 8.738 for the high-level Ki-67 tertile (95% CI: 4.286–17.816), with the low-level Ki-67 tertile used as a reference. The HR of the Ki-67 linear value was 1.033 (95% CI: 1.023–1.043).

### Response and Ki-67

**Figure 1:**
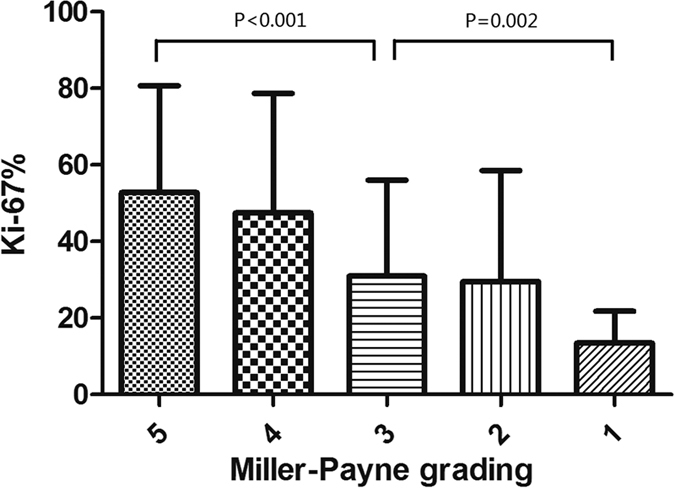
The relationship between the Ki-67 values before chemotherapy and the Miller-Payne (MP) grades. The mean Ki-67 values of the tumors were 52.8% for MP 5, 47.4% for MP 4, 30.9% for MP 3, 29.5% for MP 2 and 13.5% for MP 1.

The clinical and pathological treatment responses of the Ki-67 groups are shown in Table 2. The clinical response to the NCT was evaluated according to the RECIST 1.1 criteria. The regression of the primary tumor was assessed using the MP grades. The residual disease involvement of lymph nodes in surgical specimens was also assessed. There were statistically significant differences among the low-level, median-level, and high-level Ki-67 groups with respect to the clinical response (P < 0.001), MP grades (P < 0.001), and node status (P < 0.001). Figure 1 shows the relationship between the Ki-67 values before chemotherapy and the MP grades. The mean Ki-67 values of the tumors were 52.8% for MP 5, 47.4% for MP 4, 30.9% for MP 3, 29.5% for MP 2 and 13.5% for MP 1. There was a significant difference between a complete response and a partial response (MP 5 vs. MP 3, P < 0.001) and between a partial response and no response (MP 3 vs. MP 1, P = 0.002).

**Figure 2:**
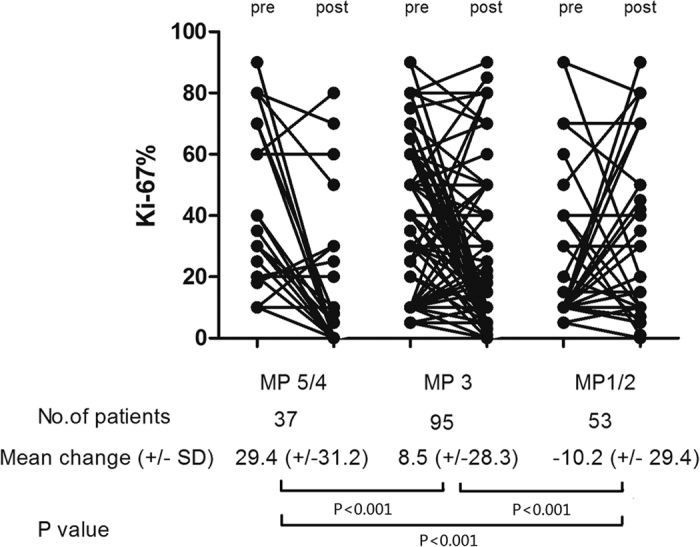
The correlation between change in Ki-67 value and Miller-Payne (MP) grades. The mean absolute Ki-67 reductions were 29.4%, 8.5%, and −10.2% in patients with an ideal response (MP 5/4), a partial response (MP 3) and a poor response (MP2/1), respectively.

Among 185 patients with a residual tumor after NCT, the median Ki-67 value significantly decreased from 20.0% before chemotherapy to 15% after chemotherapy. The correlation between the reduction of Ki-67 value and tumor regression (according to MP grades) is shown in Fig. 2. The mean absolute reduction of Ki-67 value were 29.4% in patients with an ideal response (MP 5/4), 8.5% in patients with a partial response (MP 3), and −10.2% in patients with a poor response (MP2/1) respectively. A significant reduction of Ki-67 was most likely observed in patients with a relatively better response.

### Survival and prognostic factors

The median follow-up time was 39 months. Among the 95 pCR patients, only 4 developed recurrences. However, 60 of the 185 non-pCR patients had recurrences within the follow-up period. Failure to achieve a pCR was clearly associated with worse long-term outcomes (P < 0.001).

**Figure 3:**
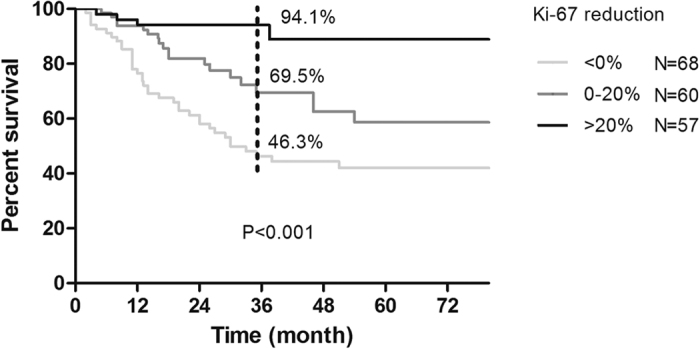
Relapse–free survival (RFS) curves by categorical Ki-67 reduction in non-pCR patients (log-rank test, P < 0.001).

In non-pCR responders, univariate survival analysis was performed to assess the prognostic value of variables. Primary tumor size (P = 0.023), residual tumor size (P < 0.001), residual node involvement (P < 0.001), MP grades (P = 0.023), vascular invasion (P = 0.027), primary tumor Ki-67 (P < 0.001), residual tumor Ki-67 (P = 0.001), and absolute reduction in Ki-67 (P < 0.001) were significant predictors of RFS and were entered into the multivariate Cox regression model with forward selection. However, patient age, menopausal status, primary node status, residual tumor grades, CK5/6 and EGFR were not significant variables. In the Cox model (shown in Table 3), the absolute change in Ki-67 value after NCT showed an independent prognostic value for RFS (HR = 0.986, 95% CI: 0.978–0.994; P = 0.001). Residual node involvement was also an independent predictor of patient outcome (HR = 0.895, 95% CI: 0.355–2.259 for 1–3 nodes and HR = 2.424, 95% CI: 1.048–5.608 for more than 4 nodes, using 0 nodes as a reference; P = 0.002). Better survival was more frequently observed in patients with a greater reduction in Ki-67 value and fewer involved nodes. The distributions of the survival curves by categorical Ki-67 reduction are shown in Fig. 3 (log-rank test, P < 0.001). All non-pCR patients were grouped by the tertile absolute reduction in Ki-67 values. The observed 3-year RFSs were 94.1%, 69.5%, and 46.3% in the three groups and demonstrated a favorable outcome in patients with a greater reduction in Ki-67 values.

## Discussion

Breast cancer that lacks ER, PR, and overexpression of HER-2, known as TNBC, is not amenable to the currently available targeted therapies and has a poor prognosis. Compared with other breast cancer subtypes, TNBC has a higher response rate to neoadjuvant chemotherapy; however, this advantage is not clearly translated into an improved overall survival^15^. This so-called TNBC paradox has attracted great attention of clinicians and researchers. Although the components are not yet clearly elucidated, it is believed that TNBC is a heterogeneous disease comprising subtypes with different biological behaviors and clinical outcomes. Gene expression analyses have identified intrinsic subtypes of the TNBC populations that are refining our understanding of breast cancer biology. A previous report showed that TNBC could be classified into 7 subtypes by gene expression microarray^16^. However, these subtypes are not well defined to enable the development of targeted therapy or prediction of treatment response. In fact, gene-expression profiling has not fully replaced classical IHC because it is not a routine practice. Thus, surrogate IHC markers have served as a more practical means of assessing preclinical and clinical predictive effects on patient outcome and differential responses to specific agents. For example, a five-marker method, which examines ER, PR, HER2, CK5/6, and the epidermal growth factor receptor (EGFR) has been proposed as a surrogate system for identifying basal-like breast cancer^17^. In our study, the majority of TNBCs (69.6%) showed the basal-like immunophenotype according to the expression of the basal markers CK 5/6 and EGFR. In some previous studies, TNBCs that expressed basal markers were associated with a significantly higher response to chemotherapy and a shorter DFS^8^,^17^, while other studies did not find any significant correlation^18^. In this study, we did not find a relationship between basal markers and treatment response or patient survival. However, the proliferative marker Ki-67 showed an important predictive and prognostic value.

It is well known that in the breast cancer phenotype system, the expression of Ki-67 has been associated with the luminal B phenotype, a high risk of relapse, and likelihood of good response to neoadjuvant chemotherapy^10^,^11^,^12^. In this study, the median Ki-67 value was 30%, which is concordant with previous studies^11^,^19^. We also confirmed that a higher Ki-67 value was correlated with better treatment response in TNBC. Among the different Ki-67 levels, there were significant differences in both clinical (a decrease in tumor size) and pathological (a reduction of tumor cells) evaluations of treatment response to NCT. Remarkably, the pCR rate was approximately 60% in patients with a primary Ki-67 value of more than 50%, which is almost quadruple the pCR rate in patients with a Ki-67 value of less than 20%. Our findings indicate that Ki-67 could further differentiate TNBCs into subtypes with different chemosensitivities.

Furthermore, we demonstrated the prognostic value of Ki-67 before and after treatment with a cohort of matched pre- and post-treatment samples. Given that the Ki-67 value was not available in excision samples from pCR patients and the survival of pCR responders had a favorable outcome, we specifically addressed the impact of Ki67 on the survival of patients who received neoadjuvant chemotherapy and did not achieve pCR. Multivariate analysis showed that the reduction in the Ki-67 value rather than the primary tumor Ki-67 value or the residual tumor Ki-67 value was a strong prognostic factor. Most previous studies have emphasized the greater significance of Ki-67 change or the residual tumor absolute Ki-67 value in the excision sample rather than the diagnostic sample^11^,^12^,^20^. These findings may be due to identifying patients in whom there remains a high chance of residual highly proliferative micrometastatic disease after neoadjuvant chemotherapy. These findings may also explain the paradox of TNBC that the high relapse among patients with residual disease is because of highly proliferative tumor biology.

Although a reduction in the absolute value of the Ki-67 change after NCT is widely observed, the NCT regimens, courses and intrinsic subtypes among the study populations are heterogeneous. The cut-off points used to define the level of Ki-67 reduction also vary in published studies. Thus, the association between the reduction of Ki-67 and prognosis has not been clearly investigated in detail. With a consistent NCT regimen of paclitaxel and platinum-containing agents administered specifically to TNBC patients, our results indicate that the reduction rate of Ki-67 was more important for the identification of patients with a high risk of relapse than Ki-67 value of the primary or residual tumor.

Interestingly, although the reduction of Ki-67 values were related with MP grades in this study (Fig. 2), we found that MP grades were not independently correlated with survival. This result is similar to the result from our former study that indicated that the reduction of cancer cells carried a poor prognostic value in ER- non-pCR patients^21^. Given that the MP grading system is mainly based on a change in cellularity during the treatment, our findings suggest that as long as residual tumor cells are present, the changes in the biology of tumors other than the proportion of reduced cells may be correlated with a survival benefit. The reduction of Ki-67 might be an effective complementary or alternative assessment to the MP grading system in the pathological evaluation of the NCT response that would further modify the determination of subsequent systemic treatment.

The main limitations of using Ki-67 values are the lack of standard measurement methods and consistent cut-off values. Routine measurements of Ki-67 have not been firmly established, and the staining method lacks analytical validity. A recent study has indicated that the evidence does not support the clinical use of Ki-67 in patients with ER-positive, Ki67-low breast cancer and 1–3 positive nodes without risk^22^. Furthermore, various cut-off values have been used among previous studies and there is no recommended consensus regarding cut-off values^23^,^24^. In St.Gallen 2013 consensus, the Panel noted that standardized cut-offs for Ki-67 have not been established and laboratory specific values should be used. Therefore, the standardization and quality assurance of laboratories in Ki-67 measurement is needed^25^. To reduce the influence of establishing cut-off points, we analyzed the predictive and prognostic value of Ki-67 as a linear variable and presented the distribution of treatment response or survival by tertile categories of Ki-67. Despite these limitations, Ki-67 measurement by IHC is still a low-cost method suitable for widespread use in clinical practice, especially in identifying the immunophenotype of TNBC.

In conclusion, our study has provided new evidence that both the biopsy Ki-67 and excision Ki-67 are important in TNBC patients who are treated with NCT according to the same protocol. The primary Ki-67 might help in further classifying TNBCs into subtypes with different responses to chemotherapy. The significant reduction of Ki-67 after treatment could indicate a favorable prognosis in non-pCR patients. Further prospective studies aimed at tailoring additional systemic treatment strategies for non-pCR patients regarding Ki-67 are warranted on a larger scale to establish Ki-67 assessment as a reliable technique in daily practice.

## Methods

### Ethics Statement

We obtained permission to collect the data from the database of Fudan University Shanghai Cancer Centre. All patients included in our study had signed informed patient consents, and our study was approved by the Ethical Committee and Institutional Review Board of Fudan University Shanghai Cancer Centre. The methods were performed in accordance with the approved guidelines.

### Study population

Between 2009 and 2014, women with large operable (primary invasive tumor >3 cm and N0-1) or locally advanced breast cancer were enrolled in a phase II study^26^ of neoadjuvant chemotherapy (NCT) using six cycles of weekly PC (paclitaxel plus carboplatin) followed by surgical resections at the Shanghai Cancer Hospital. Blood chemistry, bone scan, chest X-ray and abdominal ultrasound examination were performed to exclude metastatic disease before the initiation of NCT. The disease of all patients was confirmed to be invasive carcinoma using core needle biopsy (CNB). The node status was assessed through fine needle aspiration (FNA) of palpable lymph nodes before NCT. Patients with disease that was classified as triple-negative breast cancer (TNBC, described below) were included in this study. Patients with bilateral breast cancer, male breast cancer or inflammatory breast cancer were not included in this study.

### Treatment and response

All patients underwent six cycles of NCT with paclitaxel (80 mg/m^2^) and carboplatin (AUC 2 mg*min/ml) on days 1, 8, and 15 of a 28-day cycle. No other anti-cancer treatments, including chemotherapy, radiation therapy or endocrine therapy before surgery, were permitted. After completion of the NCT, all patients underwent mastectomy and axillary lymph node dissection (ALND) within four weeks. Subsequently, patients who failed to reach pathological complete response received three more cycles of anthracycline-containing chemotherapy. The use of radiation therapy was at the discretion of the treating radiologist and based on the disease status before NCT.

The clinical responses to NCT were evaluated based on MRI and ultrasound examinations in accordance with the response evaluation criteria in solid tumors (RECIST) version 1.1^27^. The pathological evaluation of surgical specimens was conducted at the Shanghai Cancer Hospital Pathology Center by two experienced pathologists. The Miller-Payne (MP) grading system was used to evaluate the pathological response in the breast^28^: no change or some alteration in individual malignant cells but no reduction in overall cellularity was considered as Grade 1; up to a 30% reduction of tumor cells was considered as Grade 2; between an estimated 30% and 90% reduction of tumor cells was considered as Grade 3; more than a 90% reduction of tumor cells, such that only small clusters or widely dispersed individual cells remained, was considered as Grade 4; and no remaining invasive malignant cells was considered as Grade 5. A pathological complete response (pCR) after NCT was defined as the absence of invasive carcinoma in both the breast tissue (MP Grade 5) and lymph nodes of the resected specimen (pN0).

### Immunohistochemistry

Immunohistochemistry (IHC) results were collected from the database of the pathology center. All analyses were performed on paraffin-embedded tissue sections using standard procedures for breast tumor specimens to evaluate the expression of estrogen receptor (ER), progesterone receptor (PR), human epidermal growth factor receptor-2 (HER-2), and Ki-67 before and after NCT. The cut-off values for ER positivity and PR positivity were 1% of positive tumor cells with nuclear staining. HER-2 was evaluated as 0, 1+, 2+ or 3+ using circumferential membrane-bound staining. Positivity for HER-2 (HER−2+) was considered as 3 + using IHC or as positive on fluorescence *in situ* hybridization (FISH), whereas cases with 0 to 1 + or 2 + using IHC but without FISH detection were regarded as negative for HER-2 (HER-2−). Only tumors that were ER-, PR- and HER-2− were considered as TNBC and were included in this study. The Ki-67 value was expressed as the percentage of positive cells (at least 1000) with nuclear staining in each case. CK5/6 and EGFR were considered positive if 10% or more of the tumor cells showed positive membrane expression.

### Statistical analysis

A Chi-squared test was used to evaluate the relationship between patient characteristics and the pathological response. Fisher’s exact test was performed when necessary. Multivariate logistic regression was used to identify independent predictors of pCR, with all candidate variables entering the model. The mean values shown in Figs 1 and 2 were analyzed with Student’s t test and analysis of variance (ANOVA). Relapse-free survival (RFS) was calculated from the date of surgery to the date of disease relapse (local or distant relapse or death from any cause). Patients without events or death were censored at the last follow-up. Univariate and multivariate survival analyses were performed using the Cox regression model. Survival curves were estimated using the Kaplan–Meier method, and the log-rank test was used to test for differences between the groups. All reported P-values were two-sided. P values less than 0.05 were considered significant. All analyses were performed with SPSS software (version 13.0, SPSS Company, Chicago, IL).

## Additional Information

**How to cite this article**: Wang, R.-X. *et al*. Value of Ki-67 expression in triple-negative breast cancer before and after neoadjuvant chemotherapy with weekly paclitaxel plus carboplatin. *Sci. Rep.* **6**, 30091; doi: 10.1038/srep30091 (2016).

## Figures and Tables

**Table 1:** Patient characteristics and observed pathological complete response.

Characteristics	Number of patients	Number of pCR (%)	P value	P value*
Age			0.534	0.842
<40	58	23 (39.6)		
40–59	175	58 (33.1)		
60+	47	14 (29.8)		
Menopausal status			0.779	0.948
Pre	150	52 (34.7)		
Post	130	43 (33.1)		
Tumor size at baseline			0.043	0.223
T2	136	56 (41.2)		
T3	97	27 (27.8)		
T4	47	12 (25.5)		
Node status at baseline			0.986	0.697
−	47	16 (34.0)		
+	233	79 (33.9)		
Cancer stage at baseline			0.040	0.359
II	153	60 (39.2)		
III	127	35 (27.6)		
Histology at baseline			0.102	0.433
Invasive ductal carcinoma	205	77 (37.6)		
Invasive (mixed) carcinoma	60	14 (23.3)		
Others	15	4 (26.7)		
Ki-67 expression at baseline				
Category			<0.001	<0.001
Low (<20%)	99	14 (14.1)		
Median (20%–50%)	85	25 (29.4)		
High (50%+)	96	56 (58.3)		
Linear			<0.001	<0.001
CK5/6			0.560	0.574
−	120	43 (35.8)		
+	160	52 (32.5)		
EGFR			0.168	0.372
−	131	39 (29.8)		
+	149	56 (37.6)		

*Abbreviations: pCR, pathological complete response; CK, cytokeratin; EGFR, epidermal growth factor receptor.*

*P value of the multivariate logistic regression.

**Table 2:** Clinical and pathological treatment response according to the Ki-67 tertile of the primary tumor.

Response	Ki-67 expression			P value
	low	median	high	
	n = 99 (%)	n = 85 (%)	n = 96 (%)	
*Clinical response*				
CR	13 (13.1)	20 (23.5)	40 (41.7)	<0.001
PR	52 (52.5)	43 (50.6)	39 (40.6)	
SD/PD	34 (34.3)	22 (25.9)	17 (17.7)	
*Pathological response*				
MP grades				<0.001
5/4	19 (19.3)	45 (52.9)	68 (70.8)	
3	48 (48.5)	32 (37.6)	23 (24.0)	
2/1	32 (32.3)	8 (9.4)	5 (5.2)	
Node involvement				<0.001
0	21 (21.2)	38 (44.7)	80 (83.3)	
1−3	23 (23.3)	32 (37.6)	11 (11.5)	
4+	55 (55.6)	15 (17.6)	5 (5.2)	

*Abbreviations: CR, complete response; PR, partial response; SD/PD, stable/progression disease; MP, Miller-Payne.*

**Table 3:** Variables used in the multivariate survival analysis for relapse-free survival.

Variable	RFS		
	HR	95% CI	P
Tumor size at baseline			NS
T2 vs. T3 vs. T4			
Residual tumor size (cm)			NS
0–2 vs. 2–5 vs. >5			
Residual LNs involved			0.002
0	Ref.		
1–3	0.895	0.355–2.259	
4+	2.424	1.048–5.608	
Vascular invasion			NS
Yes vs. No			
MP grades			NS
1/2 vs. 3 vs. 4/5			
Primary Ki-67			
Linear			NS
Post-NCT Ki-67			
Linear			NS
Change in Ki-67			
Linear	0.986	0.978–0.994	0.001

*Abbreviations: RFS, relapse-free survival; HR, hazard ratio; CI, confidence interval; NS, not significant; LN, lymph node; Ref, reference; MP, Miller-Payne; NCT, neoadjuvant chemotherapy.*
